# Ureteral reconstruction with appendiceal interposition graft following resection of retroperitoneal leiomyosarcoma

**DOI:** 10.1093/jscr/rjad414

**Published:** 2023-07-18

**Authors:** Taylor R Williams, Ismael Diallo, Muta Issa, Nader N Massarweh

**Affiliations:** Department of Surgery, Morehouse School of Medicine, Atlanta, GA, USA; Department of Surgery, Morehouse School of Medicine, Atlanta, GA, USA; Surgical and Perioperative Care, Atlanta Veterans Affairs Health Care System, Decatur, GA, USA; Department of Urology, Emory University School of Medicine and Veterans Affairs Medical Center, Atlanta, GA, USA; Department of Surgery, Morehouse School of Medicine, Atlanta, GA, USA; Surgical and Perioperative Care, Atlanta Veterans Affairs Health Care System, Decatur, GA, USA; Division of Surgical Oncology, Department of Surgery, Emory University School of Medicine, Atlanta, GA, USA

**Keywords:** Ureter, Ureteral reconstruction, Appendix, Appendiceal interposition graft, Retroperitoneal leiomyosarcoma

## Abstract

Ureteral defects can be repaired using a variety of different techniques that depend on the length and position of the defect. Here we describe a case where a long, upper-ureteral defect was successfully reconstructed using an appendiceal interposition graft. A 60-year-old female patient underwent resection of a right-sided retroperitoneal leiomyosarcoma that was encasing the entire upper ureter and obstructing the right kidney. The mass was resected en bloc, leaving behind an 11 cm ureteral defect. The defect was successfully reconstructed with an appendiceal interposition graft. Appendiceal interposition grafts are a feasible and effective approach for ureteral reconstruction in adults following oncologic resection. We describe various technical aspects that optimize the success of ureteral reconstruction.

## INTRODUCTION

This report describes a case involving a large, right-sided retroperitoneal leiomyosarcoma that encased the right upper third of the ureter. The encased segment of ureter was removed en bloc with the primary mass. The ureteral defect was successfully reconstructed using an appendiceal interposition graft.

## CASE PRESENTATION

A 60-year-old woman presented with a 12 cm right-sided retroperitoneal leiomyosarcoma and secondary ipsilateral hydronephrosis, managed with a right percutaneous nephrostomy tube. After discussion in multi-disciplinary tumor board, she was treated with three cycles of neoadjuvant Adriamycin and Dacarbazine. The patient responded favorably to neoadjuvant chemotherapy, evident by tumor stability on subsequent imaging. As such, resection was recommended. Her percutaneous nephrostomy tube was converted to a nephroureteral tube in preparation for surgical resection.

An open approach was undertaken to excise the mass. Upon dissection to mobilize the mass, it became apparent that the posterior aspect of the mass encased the entire upper third of the right ureter—the nephroureteral tube facilitated identification of the ureter. Ureteral resection was necessary to achieve total oncological resection with negative surgical margins. Urology was consulted intraoperatively and three management options were discussed: (i) Ligation of the right ureter with planned delayed reconstruction of the ureter, (ii) right nephrectomy and (iii) ureteral reconstruction using intestinal segment. The third option was favored given the long and healthy appearance of the appendix. As such, an en bloc resection of the mass with segmental resection of the involved ureter was performed. With the mass removed, an 11 cm gap between the superior and inferior aspect of the ureter was noted.

**Figure 1:**
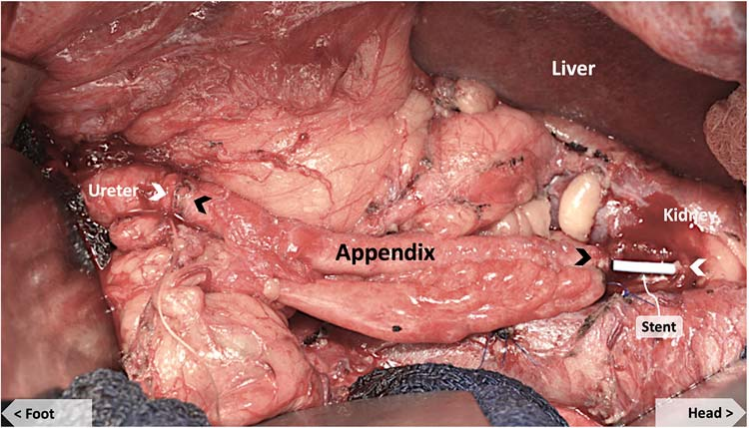
Appendiceal conduit (pre-anastomosis) showing JJ stent bridging the upper native ureter and the proximal end of the appendiceal graft. Black arrows mark the proximal and distal ends of the appendiceal graft. White arrows mark the proximal and distal ends of the native ureter.

**Figure 2:**
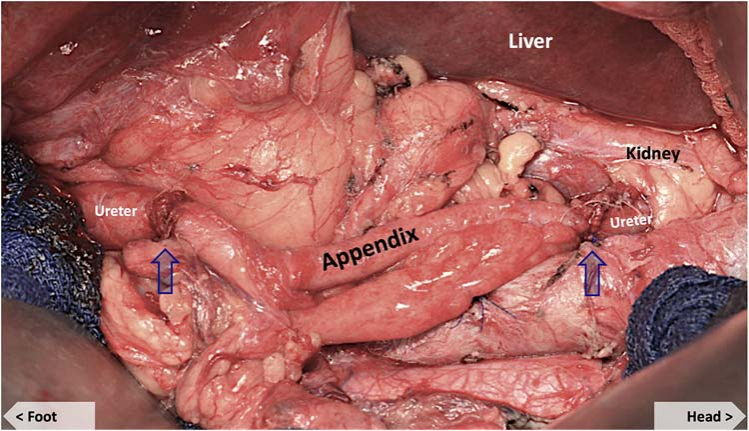
Appendiceal conduit (post-anastomosis). Arrows mark the proximal and distal uretero-appendiceal anastomosis.

The appendix was noted to have an appropriate diameter, length, location and blood supply for an interposition graft to bridge the defect. The mesoappendix was preserved and a purse-string suture was placed at the base of the appendix. The appendix was transected distal to the suture at the cecum and the appendiceal tip amputated. The appendiceal stump was cauterized and invaginated in the cecum. The appendiceal lumen was irrigated with antibiotic solution and the ends spatulated to match the size of the ureteral ends. A 6Fr × 24 cm double J ureteral stent (JJ) was from the renal pelvis, through the appendiceal graft and into the bladder, traversing the anastomoses (Fig. 1). The ureteral anastomoses were performed with interrupted 4-0 Vicryl sutures without tension (Fig. 2). A 19Fr Blake drain was placed in the retroperitoneum. The nephrostomy tube was maintained.

Postoperative recovery was uneventful. The patient was discharged on postoperative day 6. The Foley catheter and Blake drain were removed on post-op day 19. The JJ stent was removed on post-op day 39. Follow-up nephrostogram showed mild obstruction at the proximal uretero-appendiceal anastomosis. The anastomotic scar was treated successfully with endoscopic laser incision. Follow-up Mercaptuacetyltriglycerine 3 (MAG-3) nuclear renal scan at 10 months postoperatively showed normal MAG-3 renal clearance of 314 mL/min with a normal differential renal function of 47% on right and 53% on left. The patient had full recovery without long-term sequelae and remains without evidence of disease recurrence at 1 year postoperatively.

## DISCUSSION

Various surgical options are available for reconstruction of ureteral defects. Short segment defects of the proximal or mid-ureter can be repaired with a tension-free, end-to-end anastomosis. Defects of the distal ureter can be managed with a ureteroneocystostomy, with or without a Boari flap or psoas hitch [1]. For longer defects, the use of an interposition graft, as in this case, is preferred. Other options include transureteroureterostomy and renal auto-transplantation. The ideal graft should have characteristics that mimic the native ureter: capable of peristalsis; robust blood supply; optimizing drainage and minimizing absorption; preventing urine reflux; preventing stone or stricture formation [1, 2].

The first known case of ureteral reconstruction using the appendix was documented in 1912 by Melnikoff [3]. In more recent years, the appendix continues to be used for ureteral reconstruction. In children, the appendix is widely recognized as an option for lower urinary tract reconstruction, as in the appendicovesicostomy for catheterizable intestinal neo-bladder; Mitrofanoff Procedure [4]. However, the use of the appendix for ureteral reconstruction in adult oncologic resections is less studied [5]. This could be explained by the number of adult patients who have had an appendectomy.

While ureteral reconstruction can also be performed using small and large intestine, the appendix offers several advantages over other intestinal conduits. Advantages include the match in size, less absorptive surface with less metabolite absorption and a lower risk of electrolyte disorders, and avoidance of intestinal resection and anastomosis [2, 6–7]. Limitations to this operation include the finite length of the appendix and the inability to perform the operation if the appendix is absent, inflamed or damaged [7]. An appendiceal graft is more suitable for right-sided ureteral reconstruction due to its inherent ipsilateral location. The use of an appendiceal graft for left-sided ureteral reconstruction has been reported but it is less common due to its higher complication rate [6, 8]. The long-term success of appendiceal ureteral replacement in terms of satisfactory transport of urine and maintaining good renal function, has been documented [9].

There are several technical aspects that impact the outcome of an appendiceal interposition graft. An appendiceal graft is more amenable to fit in its antegrade direction, as there is less chance of torsion to the mesentery and compromise to the blood supply of the graft. Non-traumatic mobilization of the appendix and preservation of its mesentery are also important to preserve a healthy blood supply for optimal healing of anastomoses. The ends of the appendix and the native ureter are spatulated to match in size and fit to accomplish wide anastomoses. It is important to avoid electrocautery and maintain tension-free anastomoses to prevent thrombosis of blood supply and the development of anastomotic stricture. Anastomotic stricture remains the most common complication after the procedure. When it develops it tends to involve the proximal anastomosis, as it is narrower and more distant from the mesenteric blood supply. This complication can be managed with endoscopic incision using Holmium laser, as it was done in the case reported.

## CONCLUSIONS

The appendiceal interposition graft is a feasible approach to effective ureteral reconstruction in adults following oncologic resection.
